# Cryptococcal meningoencephalitis in an IgG_2_-deficient patient with multiple sclerosis on fingolimod therapy for more than five years – case report

**DOI:** 10.1186/s12883-020-01741-0

**Published:** 2020-04-27

**Authors:** Tobias Wienemann, Ann-Kristin Müller, Colin MacKenzie, Carina Bielor, Vivien Weyers, Orhan Aktas, Hans-Peter Hartung, David Kremer

**Affiliations:** 1 Institute of Medical Microbiology and Hospital Hygiene, Medical Faculty, Heinrich-Heine University Düsseldorf, 40225 Düsseldorf, Germany; 2 Department of Neurology, Medical Faculty, Heinrich-Heine University Düsseldorf, 40225 Düsseldorf, Germany

**Keywords:** Cryptococcus, Meningoencephalitis, Multiple sclerosis, Fingolimod, Blood culture

## Abstract

**Background:**

Fingolimod (Gilenya®), a first-in-class sphingosine-1-phosphate receptor modulator is approved for the treatment of relapsing-remitting multiple sclerosis. Fingolimod-induced selective immunosuppression leads to an increased risk of opportunistic infections such as cryptococcosis. So far, a total of 8 cases of fingolimod-related cryptococcal meningoencephalitis have been published.

**Case presentation:**

A 49-year-old female with relapsing-remitting multiple sclerosis presented with cephalgia, fever, confusion and generalized weakness. She had been on fingolimod therapy for the past 5.5 years. Clinical examination suggested meningoencephalitis and laboratory findings showed an IgG_2_ deficiency. Initially no pathogen could be detected, but after 4 days *Cryptococcus neoformans* was found in the patient’s blood cultures leading to the diagnosis of cryptococcal meningoencephalitis. After antimycotic therapy, her symptoms improved and the patient was discharged.

**Conclusion:**

MS patients on immunomodulatory  therapy are at constant risk for opportunistic infections. Cephalgia, fever and generalized weakness in combination with fingolimod-induced lymphopenia should be considered a red flag for cryptococcosis.

## Background

Fingolimod was approved for the treatment of relapsing-remitting multiple sclerosis (RRMS) in 2010. By sequestering lymphocytes in lymphoid tissue it prevents their access to the central nervous system. This translates clinically to a reduction of relapse rate and radiologically to a decrease of lesion load and brain volume loss. However, peripheral lymphopenia is associated with an increased risk for opportunistic infections. The ubiquitous yeast *Cryptococcus neoformans* is a well-known cause of meningoencephalitis in immunocompromised people. Between 2015 and 2019 a total of 8 cases of cryptococcal meningoencephalitis in MS patients treated with fingolimod for 2–5 years have been reported [1–8]. We now describe a case of cryptococcal meningoencephalitis diagnosed 5.5 years after therapy initiation. This is the first case of a fingolimod-treated MS patient, in which cryptococci were initially detected in blood cultures leading to the correct diagnosis. In addition, our patient suffered from IgG_2_ deficiency which is considered to be a crucial risk factor for the disease even in otherwise healthy individuals.

## Case presentation

**Fig. 1:**
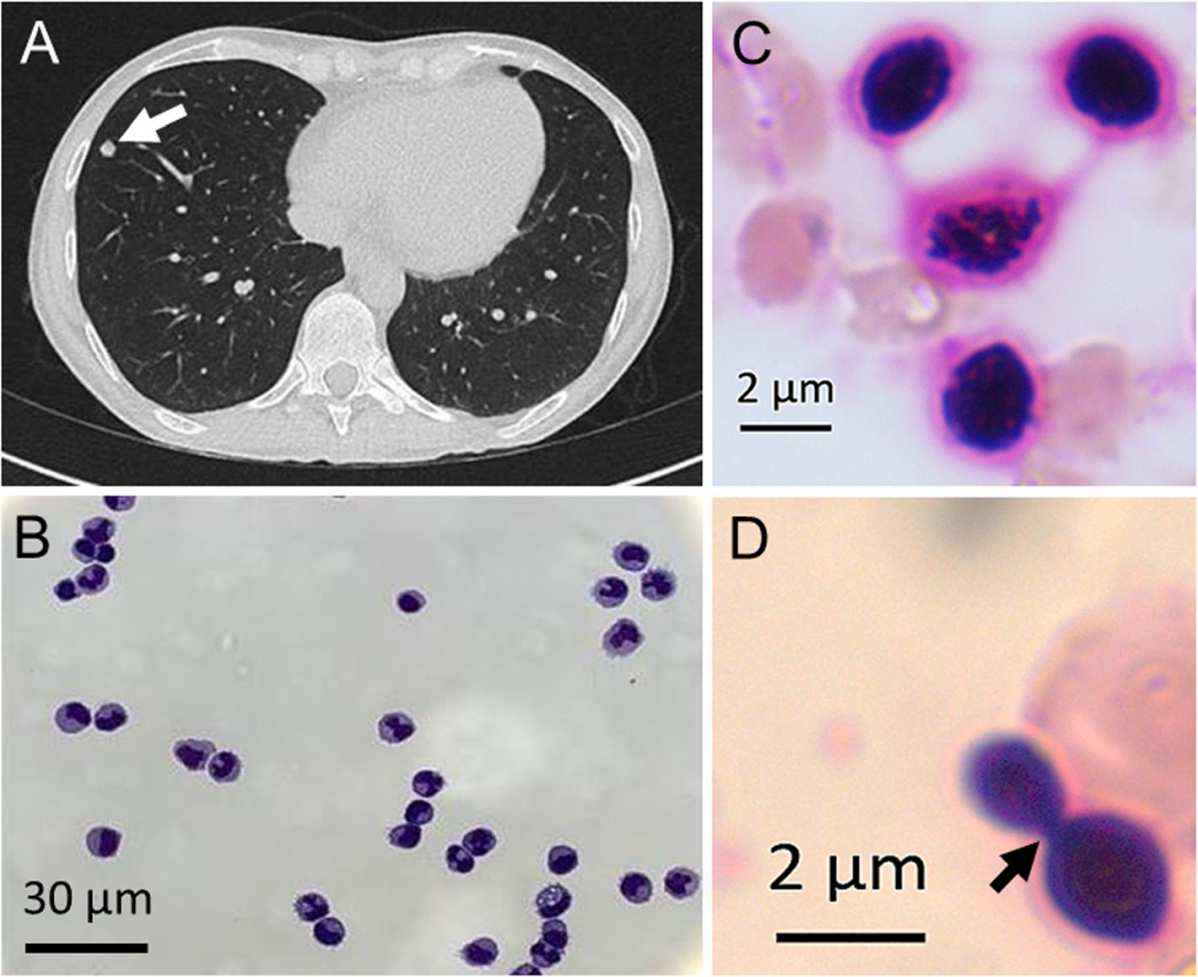
**a** Chest CT scan with lesions compatible with a pulmonary manifestation of cryptococcosis (arrow indicates lesion in the right lower lobe). **b** CSF with lympho-monocytic pleocytosis (Wright-Giemsa stain). **c***Cryptococcus neoformans* gram stain from blood culture. **d** Cryptoccocus cells featuring budding (arrow indicates the connection between mother and daughter cell) gram stain from blood culture

A 49-year-old Caucasian female with RRMS, who had been on fingolimod therapy for the past 5.5 years, presented with cephalgia, fever, confusion, coughing and generalized weakness. Prior to fingolimod, the patient had received therapies with interferons, natalizumab, mitoxantrone and rituximab which had all been discontinued due to persistent disease activity. Physical examination revealed mild temporal disorientation without new focal neurologic deficits or nuchal rigidity. Laboratory evaluation showed a normal total white cell count but a persistent absolute lymphopenia of as low as 0.09 × 1000/μl (normal range: 1.26–3.35 × 1000/μl). Chest X-ray and abdominal ultrasound yielded no signs of abnormalities. Multiple supra- and infratentorial non-gadolinium enhancing T2w lesions consistent with the diagnosis of MS were seen on MRI. There were no lesions suggestive of progressive multifocal leukoencephalopathy. Due to suspected meningoencephalitis blood cultures and a lumbar puncture were performed. Cerebrospinal fluid (CSF) analysis showed a lympho-monocytic pleocytosis of 54/μl (see Fig. 1b), elevated protein levels of 146 mg/dl and normal glucose and lactate levels. Microscopically, no pathogens were detectable. Intracranial pressure (ICP) was not elevated. The patient was diagnosed with meningoencephalitis of unclear aetiology and we started an empiric antibiotic regimen with ceftriaxone, ampicillin and acyclovir. Fingolimod therapy was stopped. CSF-PCR results for herpes and JC virus were negative and the patient remained clinically stable. Four days after admission we detected yeast-like formations in a gram stain of an aerobic blood culture bottle whereas the CSF cultures showed no signs of growth. Due to the unusual staining (Fig. 1c and d) the suspicion of *Cryptococcus* infection was raised. A lateral flow immunoassay (IMMY™) for cryptococcal antigen on the original CSF sample showed a positive antigen titre of 1:160. Sub-culture of the positive blood culture and CSF cultures demonstrated growth of *Cryptococcus neoformans*. As a result, 4 days after admission cryptococcal meningoencephalitis was diagnosed. Antimycotic susceptibility testing was conducted by microdilution. The German National Reference Centre for Invasive Fungal Infections verified the results and identified the species *Cryptococcus neoformans* var. *grubii* (serotype A) [9]. Antimycotic treatment with intravenous liposomal amphotericin B and oral fluconazole was begun. On subsequent interrogation we elicited that the patient owned a birdhouse, which she cleaned regularly. Since the pulmonary tract is the common portal of entry for *Cryptococcus* and due to the initial respiratory symptoms, we performed a chest CT scan that revealed pulmonary lesions compatible with cryptococcosis (Fig. 1a). As cryptococcus infections usually occur in severely immunocompromised individuals and since previously only 8 cases in fingolimod-treated MS patients had been reported, we performed an extensive immunological workup. Immune cell sub-analysis revealed a reduced CD4+ T helper cell count of 77/μl (normal range: 490–1640/μl) and a decreased CD8+ T suppressor cell count of 52/μl (normal range: 170–880/μl), which were to be expected in patients on fingolimod therapy. Further analysis revealed a normal total IgG level of 817 mg/dl (normal range: 700–1600 mg/dl). The levels of the IgG subclasses IgG_1_ (550 mg/dl), IgG_3_ (16.1 mg/dl) and IgG_4_ (12.2 mg/dl) also were within normal parameters. However, we found an IgG_2_ deficiency of 108 mg/dl (normal range: 169–786 mg/dl). Within 1 week of antifungal treatment the initial symptoms subsided and the CSF cell count and protein levels decreased significantly. Due to rising serum liver enzymes fluconazole was replaced by flucytosine after which liver enzymes normalized. The patient was discharged symptom-free on fluconazole monotherapy. Within an observation period of 6 months no relapse of her MS occurred after fingolimod cessation. Follow-up CSF analysis showed no abnormalities, liver enzyme levels were normal, and the absolute peripheral blood lymphocyte count had increased to 480/μl.

## Discussion and conclusion

Cryptococcal meningoencephalitis accounts for approximately 625.000 deaths per year worldwide. While most cases occur in immunocompromised individuals such as patients with AIDS, cryptococcal meningoencephalitis can also be seen in non-immunocompromised patients [10]. As in our case, *Cryptococcus neoformans* var. *grubii* is the causative agent for 95% of infections. After inhalation, cryptococcus disseminates hematogenously with a propensity for the CNS. Clinical symptoms typically include headache, fever, malaise and meningeal irritation. The most common and severe complication is an elevated ICP which occurs in more than 50% of HIV patients [9]. In non-HIV patients this complication is uncommon. Accordingly, our patient had neither clinical nor paraclinical signs of an increased ICP. CSF analysis typically shows lower cells counts in HIV patients (0–50 cells/μL) than in non-HIV patients (20–200 cells/μL). Cryptococcus can be cultured from CSF within 48 to 72 h. The unspecific clinical signs and CSF chemistry often delay diagnosis resulting in an increased morbidity and mortality [11]. Blood cultures are often positive (90%) in HIV positive patients, but rarely in non-HIV patients (21%). Our case is the first report where  blood cultures provided the right  diagnosis in a fingolimod-treated MS patient. Since blood cultures are simple to obtain and helpful to identify other CNS pathogens, their importance should be emphasised. Detection of cryptococcal antigen in CSF via lateral flow assay has a high sensitivity and specificity of more than 98%, thereby replacing the insensitive india ink staining. Discontinuation of any immunomodulatory medication and initiation of antifungal therapy is mandatory for the successful treatment of cryptococcus meningoencephalitis. Induction therapy with amphotericin B in combination with flucytosine (or fluconazole) for at least 2 weeks is recommended [11–13]. Maintenance treatment and consolidation therapy with fluconazole are advised for at least 1 year. Even though there are no official guidelines, subsequent immunomodulatory MS therapy should include simultaneous lifelong fluconazole prophylaxis. Of note, fingolimod reduces peripheral blood levels of CD4+ and CD8+ T cells by about 80% as also observed in our patient. However, this reduction is selective as targeted cell populations are naïve T cells and central memory T cells while peripheral effector memory T cells, which are key for pathogen clearance, are spared. Fingolimod also decreases the T-cell production of proinflammatory factors such as interferon γ, which is, *inter alia,* important for the defence against fungal pathogens. In addition to that, our patient suffered from an IgG_2_ deficiency. As this immunological phenomenon has so far not been described as a specific side effect of fingolimod therapy it was possibly a pre-existing condition which had unfortunately not been detected before therapy initiation. IgG_2_ is the major immunoglobulin subclass directed against polysaccharide antigens such as the cryptococcal capsular polysaccharide glucuronoxylomannan. Accordingly, IgG_2_ deficiency has been linked to *Cryptococcus gattii* meningitis in otherwise healthy non-immunocompromised patients [14]. In summary, as the other 8 cases of cryptococcal meningoencephalitis in patients on fingolimod were either fatal or lead to severe neurological disability we recommend measuring total IgG and in particular IgG_2_ levels prior to initiation of therapy in order to assess individual risk.

In summary, fever, headache and disorientation in combination with fingolimod-induced lymphopenia should always be considered a red flag for cryptococcosis and should entail lumbar puncture and a cryptococcal antigen test. Blood cultures are a valuable asset. Even though this is only the ninth reported case worldwide it underlines the necessity for constant vigilance in MS patients under immunomodulatory therapy since delayed treatment of cryptococcal meningoencephalitis is associated with increased morbidity and mortality.

## Data Availability

Not applicable.

## Abbreviations

CSF: Cerebrospinal fluid
ICP: Intracranial pressure
RRMS: Relapsing-remitting multiple sclerosis
MS: Multiple sclerosis
